# Chronotropic Incompetence in Non-Hospitalized Patients with Post-COVID-19 Syndrome

**DOI:** 10.3390/jcm10225434

**Published:** 2021-11-20

**Authors:** Amaya Jimeno-Almazán, Jesús G. Pallarés, Ángel Buendía-Romero, Alejandro Martínez-Cava, Javier Courel-Ibáñez

**Affiliations:** 1 Department of Infectious Diseases, Hospital Universitario Santa Lucía, Cartagena, 30202 Murcia, Spain; amaya.jimeno@carm.es; 2 Human Performance & Sport Sciences Laboratory, University of Murcia, 30720 Murcia, Spain; jgpallares@um.es (J.G.P.); angel.buendiar@um.es (Á.B.-R.); alejandro.mcava@gmail.com (A.M.-C.)

**Keywords:** post-COVID-19 condition, long COVID-19, long-haulers, chronic fatigue, post-exertional malaise, autonomic nervous system

## Abstract

Patients recovering from COVID-19 commonly report persistence of dyspnea, exertional fatigue, and difficulties in carrying out their daily activities. However, the nature of these symptoms is still unknown. The purpose of the study was to identify limiting causes of cardiopulmonary origin for the performance of physical exercise in post-COVID-19 condition that could explain the symptomatic persistence of dyspnea or fatigue-related symptoms. Thirty-two non-hospitalized patients with post-COVID-19 condition (i.e., still presenting a chronic symptomatic phase lasting >90 days since debut of symptoms that lasted for at least 2 months and cannot be explained by an alternative diagnosis) completed a clinical examination including echocardiography, submaximal and maximal cardiorespiratory fitness tests (Ekblom-Bak and Bruce’s protocols), and a battery of validated questionnaires about fatigue and exercise intolerance. Four participants (12.5%) reported an abnormal cardiac response to exercise during the submaximal test, which aroused suspicion of the presence of chronotropic incompetence. All of them were confirmed with a positive diagnosis maximal exercise test after cardiology screening, even with a comprehensive clinical examination, resting ECG, and echocardiogram, without other findings. No statistical differences were found in any physiological variables or questionnaire values, between patients with positive and negative diagnoses. Chronotropic incompetence and other autonomic disorders may appear in patients with mild forms of COVID-19 presentation and may persist in the long term, being responsible for exercise intolerance after resolution of acute infection. Clinicians should be aware that chronotropic incompetence and other autonomic disorders may be a complication of COVID-19 and should consider appropriate diagnostic and therapeutic interventions in these patients, especially when early exercise-related fatigability is reported.

## 1. Introduction

After a year of the coronavirus disease (COVID-19) pandemic, it has become evident how SARS-CoV2 can be responsible for damage in the central nervous system (CNS) [1] and in the autonomic nervous system (ANS), both in the acute and in the chronic, persistent phase of the disease. Concerning the persistent phase, a new emerging condition termed post-COVID-19 syndrome or post-COVID-19 condition, commonly named long-COVID-19, (i.e., persistence of clinical manifestations lasting more than 12 weeks and which cannot be explained by an alternative diagnosis [2,3]), is affecting ~10% of COVID-19 patients and merits special attention [4]. Consisting of a range of limiting symptoms which dramatically reduce quality of life, the post-COVID-19 condition patients mostly refers to fatigue, post-exertional malaise, dyspnea, headache, and many other neurocognitive conditions described as brain fog or inability to perform daily physical tasks [5].

Damage in the ANS and CNS can lead to important dysfunctions in terms of heart rate (HR), blood pressure (BP), and systemic inflammatory response [6,7]. In a large cohort of COVID-19 patients, a significant increase in mean HR followed by a decrease in mean HR was observed from the seventh day of symptoms (relative bradycardia), which was maintained until the 21st day of evolution. This alteration was associated with a loss of HR variability (HRV), both suggesting the existence of a secondary autonomic malfunction in HR control [8]. Other sequelae attributable to autonomic dysfunction have also been found in patients with long COVID-19, such as postural orthostatic tachycardia syndrome (POTS) [9]. Both direct involvement of the sinus node in the heart and injury to the regulatory centers of the brainstem have been postulated as pathogenic mechanisms responsible for poor HR control during SARS-CoV2 infection [10]. These lesions could be mediated by cytokine storm during the acute phase, by direct structural injury related to the expression of ACE II receptors (angiotensin II receptor) present in cardiac tissue, or immune-mediated by the action of specific antibodies against the brainstem and neural tissues [10].

Chronotropic incompetence is defined as a limitation to increase HR in response to the metabolic demands proposed by exercise and may be responsible for the appearance of fatigue and exercise intolerance in patients with post-COVID-19 condition [11]. During exercise, the increase in HR is mainly due to the cessation of parasympathetic activity induced by movement and, secondarily, by adrenergic sympathetic stimulation. As a result, HR increases from 30 to 50 bpm as soon as exercise begins. Likewise, when the stimulus ceases, recovery in HR occurs when vagal tone reappears. Both the alteration of the initial increase and the absence of recovery are related to exercise intolerance and poor cardiovascular prognosis in diseases such as heart failure or COPD (chronic obstructive pulmonary disease) [12].

The purpose of the study was to identify limiting causes of cardiopulmonary origin for the performance of physical exercise in post-COVID-19 condition that could explain the symptomatic persistence of dyspnea or fatigue-related symptoms commonly referred by this population.

## 2. Material and Methods

### 2.1. Experimental Design

This cross-sectional study examined the RECOVE cohort including non-hospitalized post-COVID-19 patients (NCT04718506) [13]. After clinical screening, participants completed the Ekblom-Bak submaximal cycle ergometer test under medical supervision to identify abnormalities in cardiovascular response. When chronotropic incompetence was suspected, on a second visit, participants completed Bruce’s protocol on a treadmill test for an expert cardiologist to confirm the diagnosis.

### 2.2. Participants

Participants were recruited for the study after they expressed interest on the registration website [14]. Participants originally learnt about the study through advertisements on social media or via recommendations from clinicians—mainly general practitioners and infectious diseases consultants. Thirty-two individuals fit the eligibility criteria including a diagnosis of SARS-CoV2 using real-time reverse transcriptase polymerase chain reaction (PCR) tests or antigenic rapid tests, who still presented a chronic symptomatic phase lasting >90 days since the debut of symptoms, who were not hospitalized, and who had no evidence on clinical record of pneumonia or any other organ failure related to SARS CoV-2 infection. All participants were active before the diagnosis of COVID-19, and none of them were on medication capable of interfering with HR, such as beta-blockers.

### 2.3. Echocardiography

A complete clinical examination, including electrocardiogram and echocardiography, was performed to rule out cardiovascular diseases. A resting echocardiogram was performed following standard procedures [15] by a team of expertise cardiologists. Left-ventricular (LV) systolic function was evaluated by calculating LV ejection fraction (LVEF) using the modified Simpson rule after quantification of the LV end-systolic (LVESV) and end-diastolic volumes (LVEDV) from the apical two- and four-chamber view. Right-ventricular (RV) function was assessed by measuring tricuspid annular plane systolic excursion (TAPSE) in the RV free wall. The assessment of diastolic dysfunction (DD) was conducted using pulsed Doppler, in apical four-chamber view, by registering the mitral inflow at the level of the mitral valve annulus, with the peak early diastolic velocity (E), the late diastolic velocity (A), and the assessment of the E/A ratio. The right-ventricle tricuspid annular plane systolic excursion (RV-TAPSE) was also measured.

### 2.4. Cardiorespiratory Fitness

The cardiorespiratory fitness assessment included a submaximal cycle ergometer test with HR and rate of perceived exertion (RPE). Heart rate variability (HRV) was collected for 1 week by means of the root mean square of successive differences (RMSSD) using the Welltory app [16] as a marker for autonomic nervous system response and psychological stress [17]. The submaximal test [18] consisted of two incremental, consecutive, and submaximal work rates for 4 min on a cycloergometer (Ergoline, Ergoselect 200, Bitz, Alemania). Pedal frequency was 60 revolutions per minute. The Rating Scale of Perceived Exertion from 6–20 (RPE, Borg) [19] was used, being > 16 when the test ended. Moreover, oxygen saturation (Hylogy MD-H32, Shenzhen, China), BP (Omro M2 basic, Omron Healthcare, Milton Keynes, UK), and HR were monitored prior to the exercise phase (5 min standing), during exercise (8 min submaximal test), and during the recovery phase (3 min standing).

### 2.5. Chronotropic Incompetence Diagnosis

When chronotropic incompetence was suspected, to confirm this diagnosis, patients underwent a maximal graded exercise test according to Bruce’s treadmill protocol [20]. To diagnose chronotropic incompetence, the inability to reach 80% of the age-estimated HRmax or HR reserve obtained during a maximal incremental exercise test had to be evidenced [12]. Ideally, to avoid the possibility of not reaching high HR due to low exercise capacity, the metabolic chronotropic index (MCI) can be defined by the regression line between the percentage of reserve HR and the percentage of reserve oxygen consumption. This shows that exercise limitation is mediated by the impossibility of increasing HR and not because of a poor exercise capacity [21]. MCI is calculated using the relationship among age, HR, and exercise capacity for a given stage of the maximal stress test as follows: HR stage = ((220 − age − HR rest)) × (METs stage − 1)/(METs peak − 1) + HR rest). Any single result of MCI for a stage of ≤0.8 is considered diagnostic of chronotropic incompetence [12,22].

### 2.6. Dyspnea, Fatigue, and Exercise Intolerance in Daily Living Activities

Participants were provided with a battery of self-rating questionnaires (the higher the score, the worst the health status) to evaluate dyspnea, fatigue, and exercise intolerance in daily living activities: Chalder Scale (Chalder Fatigue Scale, CFS-11) [23], Fatigue Severity Scale (FSS) [24], DePaul Symptom Questionnaire Short Form (DSQ-14 short form) [25], Post-COVID-19 Functional Status (PCFS) scale [26], and Modified Medical Research Council Dyspneal Scale (mMRC) [27].

### 2.7. Statistical Analysis

Descriptive data analysis included means and standard deviations. Crosstabs and chi-squared analysis were used to examine the distribution of symptoms between people with positive and negative chronotropic incompetence diagnoses. Mean differences were identified by Student’s *t*-test for independent samples. Calculations were performed using IBM SPSS v. 20.0 (Armonk, NY, USA: IBM Corp.).

## 3. Results

**Figure 1:**
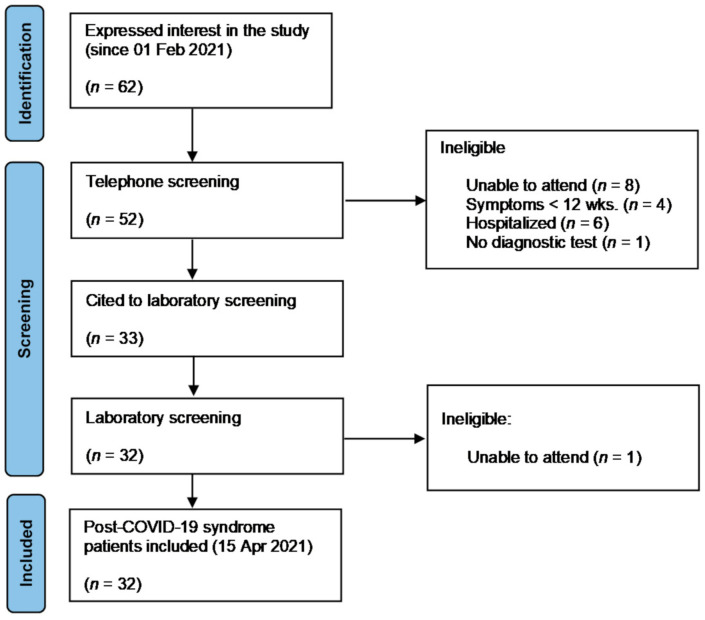
PRISMA flow diagram of recruitment results.

From 62 patients who expressed interest in the study from 1 February to 15 April, 32 complied with inclusion/exclusion criteria and were enrolled in the study (Figure 1). These thirty-two individuals presented a chronic symptomatic phase lasting >90 days since debut of symptoms, were not hospitalized, and had no evidence on clinical record of pneumonia or any other organ failure related to SARS CoV-2 infection. SARS-CoV2 infection was diagnosed using real-time reverse transcriptase polymerase chain reaction (PCR) tests or antigenic rapid tests. All participants were active before the diagnosis of COVID-19, and none of them were on medication capable of interfering with HR, such as beta-blockers.

Baseline characteristics of participants are shown in Table 1. Participants had a mean age of 45 years and were mostly female (69%). The most commonly reported pre-existing conditions were psychiatric history, asthma, and hypertension. There were no abnormalities in terms of heart rhythm, heart rate, PR interval, QRS interval, and QTc (corrected QT interval), nor were there repolarization abnormalities in the resting ECG.

**Figure 2:**
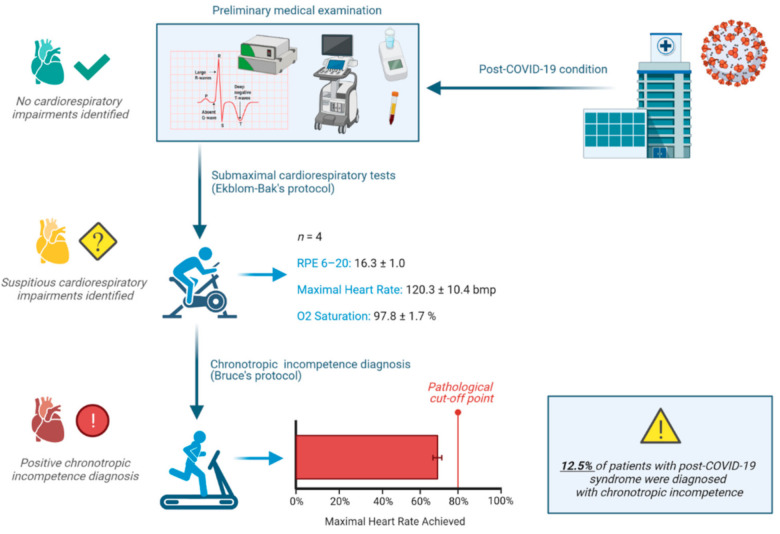
Infographic showing the experimental design and main findings.

Four participants (12.5%) reported an abnormal cardiac response to exercise during the submaximal test, i.e., HRmax ≤ 70% of estimated HRmax with an elevated RPE > 16 (6–20 scale) which aroused suspicion of the presence of chronotropic incompetence. To confirm this diagnosis, patients underwent a maximal graded exercise test according to Bruce’s treadmill protocol. Not one of them was able to reach 80% of the age-estimated HRmax (M ± SD = 69.6% ± 5.0%); therefore, chronotropic incompetence was diagnosed. Comprehensive clinical examination and resting ECG and echocardiography parameters were withing normal limits (Table 2). Likewise, no statistical differences were found in echocardiography variables or in exercise intolerance and fatigue questionnaires between patients with positive and negative diagnoses (Table 3). The experimental design and main findings are depicted in Figure 2.

## 4. Discussion

In this study, we examined a cohort of non-hospitalized patients with post-COVID-19 condition to identify a possible explanation for the exercise intolerance and fatigue, commonly referred by this population. The results showed the presence of chronotropic incompetence in 12.5% of ambulatory patients as a possible central cause for these symptoms and not only due to skeletal muscle dysfunction as other have suggested [28]. To our knowledge, this is one of few studies [11,28,29] exploring functional cardiorespiratory abnormalities in post-COVID-19 conditions and the first to describe the long-term chronotropic incompetence persistence in a non-hospitalized post-COVID-19 syndrome cohort.

The presence of autonomic dysfunction, understood as increased sympathetic activity and loss of parasympathetic counter-regulation that characterizes other cardiovascular diseases and the hyperacute phase of COVID-19 [8,30], may not be an expected long-term finding. It is possible that, because of sympathetic overstimulation in the early stages of the SARS-CoV2 infection, there is a depletion of β adrenergic receptors in the heart and, thus, a loss of tachycardia in response to the demand proposed by exercise, as occurs, for example, in heart failure or COPD [21]. It could also be the case that the second phase of HR activation upon exercise, mediated by the sympathetic system, disappears due to direct or immune-mediated damage to the ANS during the COVID-19. The direct effect of the difficulty to increase HR, adapting it to the demand of exercise, is the inability to increase cardiac output. Therefore, peripheral muscle perfusion decreases, determining the sensation of early fatigue and dyspnea during exercise.

A new contribution of the present study is the use of a submaximal, safe, short, reproducible, and noninvasive test (Ekblom-Bak protocol) for the identification of patients with exercise limitations and may assist in the identification of cardiac complications such as chronotropic incompetence. As a practical application, we found that people achieving HRmax ≤ 70% of estimated HRmax with an elevated RPE > 16 (6–20 scale) were likely to get a positive diagnosis. In fact, this finding is clinically relevant in individuals with post-COVID-19 condition complaining of dyspnea and post-exercise malaise or fatigue, because basing intensities of exercise on predicted maximal HR could be nefarious. This would lead to an overestimation of the target heart rate during exercise, whereby the subsequent inability to achieve it will cause discomfort, as well as a sensation of shortness of breath, and, in the short term, compromise adherence to the training program. Given that the Ekblom-Bak protocol is submaximal in nature, we can also conclude that the limitation of exercise appears not only at high intensity, but also at moderate intensities, which clearly implies the commitment of the activities of daily life. In addition, the use of adapted exercise tests as a screening tool in people with post-COVID-19 condition seems important considering that spirometry may fail in identifying exertional intolerance and dyspnea in this population [31]. The ongoing RECOVE study [13] can provide information about best practice for prescribing and monitoring exercise intensity in post-COVID-19 condition and whether training could normalize the chronotropic response in these subjects.

The persistent clinical manifestations of post-COVID-19 condition are extraordinarily varied and multisystemic in nature [5]. Fatigue and malaise are the most common symptoms along with dyspnea, and all of them can be related to cardiopulmonary disorders and other underlying conditions which should ideally be excluded to confirm the diagnosis of post-COVID-19 condition [32,33]. Nonetheless, it should be noted that, since fatigue is a common manifestation of many diseases and does not have a gold standard to confirm its diagnosis, the attribution of the symptom may vary across individuals and may be influenced by other neuropsychological or social disturbances.

HRV has previously been used in patients with acute COVID-19 as a noninvasive measure of autonomic function, finding that, after an early increase in parasympathetic activity, patients with elevations of poor prognostic biomarkers, such as CRP (C-reactive protein), exhibited a prior loss of HRV [34,35]. This implies that HRV determination may have prognostic implications. To date, the RECOVE study includes the only known data on the standardized registry of HRV in patients with post-COVID-19 condition (unpublished data). In chronotropic incompetent patients, we found high rMSSDs, in the time-domain measures of HRV, which suggest that, in the persistent phase, parasympathetic activity predominates, which could explain the insufficient heart rate response during exercise and impaired wellbeing perception [36]. The effect of anxiety, depression, stress, loss of quality of life, and deconditioning on these findings is unknown.

It must be noted that the current findings are limited by the reduced sample size, the lack of a control group, and the absence of previous stress tests. We cannot rule out the possibility that failure to achieved estimated HR is justified by COVID-19 alone. Nonetheless, this study is strengthened by the novelty of the findings and its practical implications (i.e., the use of the 8 min Ekblom-Bak test to screen for cardiovascular abnormalities) for clinicians dealing with patients with post-COVID-19 conditions or other conditions characterized by unexplained fatigue and exercise intolerant symptoms.

## 5. Conclusions

Chronotropic incompetence and other autonomic disorders may appear in patients with mild forms of COVID-19 presentation and persist in the long term, being responsible for exercise intolerance after resolution of the acute infection. Clinicians should be aware that chronotropic incompetence and other autonomic disorders may be a frequent complication of COVID-19 and should consider an appropriate diagnostic test, especially when early exercise-related fatigability is reported. Our research group supports that, in those patients in whom structural damage caused by SARS-CoV2 infection has been ruled out, a stress test should be performed to complete the diagnosis. Carrying out an 8 min Ekblom-Bak test is a simple, accurate, reproducible, non-time-consuming, and low-risk tool, in any clinical setting, including primary care. The presence of simple criteria (HRmax ≤70% of estimated HRmax with an elevated RPE > 16 (6–20 scale)) can indicate the diagnosis of chronotropic incompetence. Once confirmed by ergospirometry, the decision on the prescription of physical exercise must be individualized. The direct effects of training on the reversal of chronotropic incompetence are not well known; thus, new studies in people with post-COVID-19 condition must be carried out.

## Figures and Tables

**Table 1:** Characteristics, clinical history, and symptomatology of recruited patients with post-COVID-19 syndrome (*n* = 32).

Variable		Variable	
Age (years)	44.7 ± 10.9	Total mean symptoms (*n*)	6.8 ± 3.3
Sex (*n*)		Symptom’s length (weeks)	23.1 ± 13.6
Male	10 (31.3)	Symptoms	
Female	22 (68.8)	Fatigue	26 (81.3)
Body composition		Dyspnea	18 (56.3)
Body mass (kg)	72.3 ± 14.8	Lack of concentration	18 (56.3)
Height (m)	1.66 ± 0.10	Memory problems or confusion	17 (53.1)
BMI (kg·m^−2^)	26.0 ± 4.4	Low mood	17 (53.1)
Fat mass (%)	30.6 ± 8.3	Brain fog	17 (53.1)
Lean body mass (kg)	49.9 ± 11.6	Insomnia or sleep disturbances	17 (53.1)
Comorbidity (*n*)		Headache	13 (40.6)
Psychiatric conditions	12 (37.5)	Myalgia	10 (31.3)
Asthma	5 (15.6)	Anxiety	10 (31.3)
Hypertension	2 (6.3)	Loss of smell/taste	9 (28.1)
Structural heart disease	2 (6.3)	Hair loss	8 (25.0)
COPD	1 (3.1)	Chest pain	8 (25.0)
Diabetes	1 (3.1)	Dizziness	7 (21.9)
Toxic habits (*n*)		Low-grade fever	7 (21.9)
Alcohol	3 (9.4)	Palpitations	5 (15.6)
Active smoker	2 (6.3)	Weight loss	5 (15.6)
Former smoker	9 (28.1)	Cough	4 (12.5)
Medication (*n*)		Diarrhea	4 (12.5)
Taking medication	25 (78.1)	Abdominal pain	3 (9.4)
Antidepressants	13 (40.6)	Loss appetite	3 (9.4)
Benzodiazepines	11 (34.4)	Nausea and/or vomiting	2 (6.3)
Bronchodilators	7 (21.9)	Evolution	
		Fluctuating course	19 (59.4)
		Progressive improvement	23 (71.9)

Data are means and standard deviations (M ± SD) or frequencies and percentages (*n* (%)). COPD: chronic obstructive pulmonary disease.

**Table 2:** Patients with post-COVID-19 syndrome and chronotropic incompetence.

Age, Sex	Main Symptoms	Symptoms (Length in Weeks)	HRV-RMSSD (ms)	Estimated HRmax (bpm)	Test HRmax (bpm)	CI (% HRmax)
52, female	Cephalea, mental fog, cognitive impairment, anosmia, ageusia, dyspnea	9 (20)	55.1	173	102	Positive (62.1)
30, male	Cephalea, anosmia/dysgeusia, dyspnea, fatigue	7 (17)	75.9	186	121	Positive (71.0)
50, male	Mental fog, anosmia/dysgeusia, fatigue	3 (18)	82.4	174	126	Positive (72.9)
47, male	Dyspnea, fatigue	2 (12)	32.4	177	128	Positive (72.3)

HRV: heart rate variability. RMSSD: root mean square of successive differences, mean values from 1 week records. Estimated HRmax: estimated maximal heart rate from standardized equations. Test HRmax: maximal heart rate achieved in the Ekblom-Bak test registered by HR monitor. CI: chronotropic incompetence.

**Table 3:** Patients with post-COVID19 syndrome and chronotropic incompetence.

Variable	All	Positive Chronotropic Diagnosed	Negative Chronotropic Diagnosed	Sig. (*p*)
Echocardiography				
LVEF (%)	62.7 ± 3.7	59.7 ± 2.6	63.1 ± 3.7	0.070
RV-TAPSE (mm)	22.9 ± 2.3	23.5 ± 2.7	22.8 ± 2.3	0.671
LVEDV (cm^3^/m^2^)	42.8 ± 5.0	42.1 ± 4.6	42.9 ± 5.1	0.769
E/A (cm·s^−1^)	1.2 ± 0.4	1.2 ± 0.4	1.1 ± 0.4	0.936
Fatigue and exercise intolerance				
CFQ-11 Likert	21.9 ± 7.3	20 ± 13.1	22.1 ± 6.7	0.805
CFQ-11 bimodal	7.7 ± 3.0	6.6 ± 3.5	7.8 ± 2.9	0.612
FSS	5.3 ± 1.2	6.2 ± 1.3	5.2 ± 1.2	0.344
DSQ-14 frequency	30.4 ± 8.9	33.6 ± 7.2	30.1 ± 9.1	0.498
DSQ-14 severity	26.1 ± 9.3	32.6 ± 10.0	25.4 ± 9.1	0.209
DSQ-14 0–100	54.8 ± 20.2	49.7 ± 35.8	55.6 ± 18.0	0.597
PCFS	2.4 ± 1.0	1.5 ± 0.7	2.5 ± 1.0	0.162
mMRC	1.3 ± 0.9	2.0 ± 1.0	1.3 ± 0.9	0.361

LVEF: left-ventricular ejection fraction, RV-TAPSE: right-ventricular tricuspid annular plane systolic excursion, LVEDV: left-ventricular end-diastolic volume, E/A ratio: early diastolic velocity (E), late diastolic velocity (A). CFQ-11: Chalder Fatigue Scale. FSS: Fatigue Severity Scale. DSQ-14: The DePaul Symptom Questionnaire. PCFS: Post-COVID-19 Functional Status Scale. mMRC: Modified Medical Research Council dyspnea scale.

## Data Availability

The data presented in this study are available on request from the corresponding author. The data are not publicly available due to privacy reasons.

## References

[1] Lu Y., Li X., Geng D., Mei N., Wu P.Y., Huang C.C., Jia T., Zhao Y., Wang D., Xiao A. (2020). Cerebral Micro-Structural Changes in COVID-19 Patients—An MRI-based 3-month Follow-up Study: A brief title: Cerebral Changes in COVID-19. EClinicalMedicine.

[2] WHO Headquarters (HQ). A Clinical Case Definition of Post COVID-19 Condition by a Delphi Consensus 2021. https://apps.who.int/iris/rest/bitstreams/1376291/retrieve.

[3] NICE (National Institute for Health and Care Excelence) COVID-19 Rapid Guideline: Managing the Long-Term Effects of COVID-19; 18 December 2020. https://pathways.nice.org.uk/.

[4] UK Office for National Statistics (2021). Prevalence of Ongoing Symptoms Following Coronavirus (COVID-19) Infection in the UK: 1 April 2021.

[5] Jimeno-Almazán A., Pallarés J.G., Buendía-Romero Á., Martínez-Cava A., Franco-López F., Sánchez-Alcaraz Martínez B.J., Bernal-Morel E., Courel-Ibáñez J. (2021). Post-covid-19 syndrome and the potential benefits of exercise. Int. J. Environ. Res. Public Health.

[6] Goldberger A.L.S.P. UpToDate. Evaluation of Heart Rate Variability. Last Update 24 January 2020. Review 23 May 2021. https://www.uptodate.com/contents/evaluation-of-heart-rate-variability.

[7] Zhao M., Sun L., Liu J.J., Wang H., Miao Y., Zang W.J. (2012). Vagal nerve modulation: A promising new therapeutic approach for cardiovascular diseases. Clin. Exp. Pharmacol. Physiol..

[8] Natarajan A., Su H.W., Heneghan C. (2020). Assessment of physiological signs associated with COVID-19 measured using wearable devices. Npj. Digit. Med..

[9] Dani M., Dirksen A., Taraborrelli P., Torocastro M., Panagopoulos D., Sutton R., Lim P.B. (2021). Autonomic dysfunction in ‘long COVID’: Rationale, physiology and management strategies. Clin. Med. J. R. Coll. Physicians Lond..

[10] Buchhorn R., Willaschek C., Baumann C. (2021). SARS-CoV-2 infections and the autonomic nervous system. Monatsschr. Kinderheilkd..

[11] Szekely Y., Lichter Y., Sadon S., Lupu L., Taieb P., Banai A., Sapir O., Granot Y., Hochstadt A., Friedman S. (2021). Cardiorespiratory Abnormalities in Patients Recovering from Coronavirus Disease 2019. J. Am. Soc. Echocardiogr..

[12] Brubaker P.H., Kitzman D.W. (2011). Chronotropic incompetence: Causes, consequences, and management. Circulation.

[13] Courel-Ibáñez J., the RECOVE Group Rehabilitation for Post-COVID-19 Syndrome through a Supervised Exercise Intervention: The RECOVE Project [NCT04718506] 2021. NCT04718506.

[14] Human Performance & Sports Science. http://www.hpsportsscience.com/recove.

[15] Mitchell C., Rahko P.S., Blauwet L.A., Canaday B., Finstuen J.A., Foster M.C., Horton K., Ogunyankin K.O., Palma R.A., Velazquez E.J. (2019). Guidelines for Performing a Comprehensive Transthoracic Echocardiographic Examination in Adults: Recommendations from the American Society of Echocardiography. J. Am. Soc. Echocardiogr..

[16] Tyapochkin K., Kovaleva M., Smorodnikova E., Pravdin P. (2020). Smartphone App Stress Assessments: Heart Rate variability vs Perceived Stress in a Large Group of Adults. medRxiv.

[17] Thomas B.L., Claassen N., Becker P., Viljoen M. (2019). Validity of Commonly Used Heart Rate Variability Markers of Autonomic Nervous System Function. Neuropsychobiology.

[18] Björkman F., Ekblom-Bak E., Ekblom Ö., Ekblom B. (2016). Validity of the revised Ekblom Bak cycle ergometer test in adults. Eur. J. Appl. Physiol..

[19] Borg G. (1970). Perceived exertion as an indicator of somatic stress. Scand. J. Rehabil. Med..

[20] Pollock M.L., Bohannon R.L., Cooper K.H., Ayres J.J., Ward A., White S.R., Linnerud A.C. (1976). A comparative analysis of four protocols for maximal treadmill stress testing. Am. Heart J..

[21] Hulo S., Inamo J., Dehon A., Le Rouzic O., Edme J.L., Neviere R. (2016). Chronotropic incompetence can limit exercise tolerance in COPD patients with lung hyperinflation. Int. J. COPD.

[22] Engeseth K., Hodnesdal C., Grundvold I., Liestøl K., Gjesdal K., Kjeldsen S.E., Erikssen J.E., Bodegard J., Skretteberg P.T. (2016). Temporal reduction in chronotropic index predicts risk of cardiovascular death among healthy middle-aged men: A 28-year follow-up study. J. Am. Heart Assoc..

[23] Jackson C. (2015). The Chalder Fatigue Scale (CFQ 11). Occup. Med. (Chic Ill).

[24] Krupp L.B., Larocca N.G., Muir Nash J., Steinberg A.D. (1989). The Fatigue Severity Scale: Application to Patients with Multiple Sclerosis and Systemic Lupus Erythematosus. Arch. Neurol..

[25] Jason L.A., So S., Brown A.A., Sunnquist M., Evans M. (2015). Test–retest reliability of the DePaul Symptom Questionnaire. Fatigue Biomed Health Behav..

[26] Klok F.A., Boon G.J.A.M., Barco S., Endres M., Miranda Geelhoed J.J., Knauss S., Rezek S.A., Spruit M.A., Vehreschild J., Siegerink B. (2020). The Post-COVID-19 Functional Status Scale: A Tool to Measure Functional Status over Time after COVID-19. Eur. Respir. J..

[27] Mahler D.A., Wells C.K. (1988). Evaluation of Clinical Methods for Rating Dyspnea. Chest.

[28] Singh I., Joseph P., Heerdt P.M., Cullinan M., Lutchmansingh D.D., Gulati M., Possick J.D., Systrom D.M., Waxman A.B. (2021). Persistent Exertional Intolerance After COVID-19: Insights from Invasive Cardiopulmonary Exercise Testing. Chest.

[29] Baratto C., Caravita S., Faini A., Perego G.B., Senni M., Badano L.P., Parati G. (2021). Impact of COVID-19 on exercise pathophysiology: A combined cardiopulmonary and echocardiographic exercise study. J. Appl. Physiol..

[30] Del Rio R., Marcus N.J., Inestrosa N.C. (2020). Potential Role of Autonomic Dysfunction in Covid-19 Morbidity and Mortality. Front Physiol..

[31] Lam G.Y., Befus A.D., Damant R.W., Ferrara G., Fuhr D.P., Stickland M.K., Varughese R.A., Wong E.Y., Smith M.P. (2021). Exertional Intolerance and Dyspnea with Preserved Lung Function: An Emerging Long COVID Phenotype?. Respir. Res..

[32] Nasserie T., Hittle M., Goodman S.N. (2021). Assessment of the Frequency and Variety of Persistent Symptoms among Patients with COVID-19: A Systematic Review. JAMA Netw. Open.

[33] Iqbal F.M., Lam K., Sounderajah V., Clarke J.M., Ashrafian H., Darzi A. (2021). Characteristics and predictors of acute and chronic post-COVID syndrome: A systematic review and meta-analysis. EClinicalMedicine.

[34] Kaliyaperumal D., Rk K., Alagesan M., Ramalingam S. (2021). Characterization of Cardiac Autonomic Function in COVID-19 Using Heart Rate Variability: A Hospital Based Preliminary Observational Study. J. Basic Clin. Physiol. Pharmacol..

[35] Hasty F., García G., Dávila H., Wittels S.H., Hendricks S., Chong S. (2021). Heart Rate Variability as a Possible Predictive Marker for Acute Inflammatory Response in COVID-19 Patients. Mil. Med..

[36] Bourdillon N., Yazdani S., Schmitt L., Millet G.P. (2020). Effects of COVID-19 Lockdown on Heart Rate Variability. PLoS ONE.

